# SLCO1B1 Polymorphisms and Statin-Induced Myopathy

**DOI:** 10.1371/currents.eogt.d21e7f0c58463571bb0d9d3a19b82203

**Published:** 2013-12-04

**Authors:** Alison Stewart

**Affiliations:** McKing Consulting Corp., Atlanta, Georgia, USA; Centers for Disease Control and Prevention, Atlanta, Georgia, USA

## Abstract

Statin drugs are highly effective in lowering blood concentrations of LDL-cholesterol, with concomitant reduction in risk of major cardiovascular events. Although statins are generally regarded as safe and well-tolerated, some users develop muscle symptoms that are mostly mild but in rare cases can lead to life-threatening rhabdomyolysis. The SEARCH genome-wide association study, which has been independently replicated, found a significant association between the rs4149056 (c.521T>C) single-nucleotide polymorphism (SNP) in the SLCO1B1 gene, and myopathy in individuals taking 80 mg simvastatin per day, with an odds ratio of 4.5 per rs4149056 C allele. The purpose of this paper is to assemble evidence relating to the analytical validity, clinical validity and clinical utility of using SLCO1B1 rs4149056 genotyping to inform choice and dose of statin treatment, with the aim of minimising statin-induced myopathy and increasing adherence to therapy. Genotyping assays for the rs4149056 SNP appear to be robust and accurate, though direct evidence for the performance of array-based platforms in genotyping individual SNPs was not found. Using data from the SEARCH study, calculated values for the clinical sensitivity, specificity, positive- and negative-predictive values of a test for the C allele to predict definite or incipient myopathy during 5 years of 80 mg/day simvastatin use were 70.4%, 73.7%, 4.1% and 99.4% respectively. There is a need for studies comparing the clinical validity of SLCO1B1 rs4149056 genotyping with risk scores for myopathy based on other factors such as racial background, statin type and dose, gender, body mass index, co-medications and co-morbidities. No direct evidence was found for clinical utility of statin prescription guided by SLCO1B1 genotype.

## Clinical scenario

Statins are 3-hydroxy-3-methylglutaryl coenzyme A (HMG-CoA) reductase inhibitors which reduce the risk of coronary events and death by lowering blood concentrations of low density lipoprotein cholesterol (LDL-c).^1^^,^ 2 ^,^ 3 ^,^ 4 They are generally regarded as safe and well tolerated. However, some patients experience adverse muscle symptoms; these are mostly relatively mild but, in rare cases, severe muscle damage with renal failure (rhabdomyolysis) may occur (reviewed in 5 ^,^ 6). Recently, an autoimmune form of necrotising myositis has also been identified as a rare statin-associated myopathy (reviewed in 7).

There are no standard definitions or terminology for statin-associated muscle symptoms (Table 1 8 ^,^ 9 ^,^ 10 ^,^ 11 ^,^ 12 ^,^ 13), an issue that complicates attempts to establish the incidence of the condition. There is also uncertainty about the relationship between clinical symptoms, and levels of biochemical markers of muscle toxicity such as serum creatine kinase (CK). Most myopathy reported by statin users takes the form of myalgia: muscle ache, tenderness or weakness without elevated CK. Biochemically confirmed myopathy is generally associated with CK levels at least 10 times the upper limit of normal (ULN). Some classifications include an intermediate phenotype (“incipient” myopathy in the large SEARCH study,^8^ myositis in the ACC/AHA/NHLBI classification,^10^ hyperCKemia in the Canadian Working Group consensus,^13^ “mild myopathy” or “mild intolerance” in some other studies) with CK levels generally between 3× and 10×ULN, with or without muscle symptoms, and, in some cases, an additional criterion of elevated serum alanine aminotransferase (AAT).^8^ Rhabdomyolysis is diagnosed by CK levels substantially above 10×ULN (>50×ULN in the FDA classification), together with symptoms such as brown urine, elevated serum creatine and evidence of organ damage. Despite the inclusion of CK levels in most diagnostic criteria for myopathy, there is some evidence that clinically significant muscle toxicity can occur without increase in CK levels.^14^

**Table 1. Definitions of statin-induced myopathy:** 

Source	Terms used	Definitions
**ACC/AHA/NHBLI ** 10	Myopathy	Any disease of muscle
	Myalgia	Muscle ache or weakness, no CK elevation
	Myositis	Muscle symptoms with increased CK
	Rhabdomyolysis	Muscle symptoms, CK substantially >10×ULN, creatine elevation (usually with brown urine and urinary myoglobin)
**National Lipid Association ** 11	Myopathy	Myalgia (muscle pain or soreness), weakness and/or cramps, plus CK>10×ULN
	Rhabdomyolysis	CK>10,000 IU/L or CK>10×ULN plus elevated serum creatine or intervention with intravenous hydration
**FDA ** 12	Myopathy	CK≥10×ULN
	Rhabdomyolysis	CK>50×ULN and organ damage e.g. renal failure
**SEARCH Study ** 8 ^,^ 9	Incipient myopathy	CK>3×ULN and >5× baseline plus AAT≥1.7× baseline, with or without muscle symptoms
	Definite myopathy	CK>10×ULN with muscle symptoms
	Rhabdomyolysis	CK>40×ULN plus evidence of end-organ damage e.g. creatine elevation
**Canadian Working Group Consensus ** 13	Myopathy	Any disease of muscle
	Symptomatic myopathy	Muscle ache/weakness
	Myalgia	CK≤ULN
	Myositis	CK>ULN
	Rhabdomyolysis	CK≥10×ULN (potential for renal dysfunction; need for hydration therapy)
	HyperCKemia	
	Mild, grade 1	CK>ULN,≤5×ULN; may/may not have myositis
	Mild, grade 2	CK>5×ULN,≤10×ULN; may/may not have myositis
	Moderate	CK>10×ULN,≤50×ULN; may/may not have rhabdomyolysis, with/without renal dysfunction
	Severe	CK>50×ULN; may/may not have rhabdomyolysis, with/without renal dysfunction

Clinical trials and other large studies of standard-dose statin monotherapy have generally found a low frequency of statin-associated muscle toxicity, with definite myopathy (CK>10×ULN with muscle symptoms) in about 0.01%-0.3% of patients, and rhabdomyolysis in approximately 0.003%-0.01%.^9^^,^ 15 ^,^ 16 ^,^ 17 Meta-analyses of randomised controlled trials have found no significant difference between those on statin and those on placebo.^18^^,^ 19 ^,^ 20 ^,^ 21 Rates of severe myopathy are also low in reports to drug-monitoring authorities.^22^^,^ 23 The frequency of mild or moderate muscle symptoms varies widely in different reports and has tended to be significantly higher in observational studies than in clinical trials (5%-20%, depending on dose).^24^^,^ 25 ^,^ 26 Some commentators (e.g. 5 ^,^ 27)claim that results from clinical trials do not accurately reflect the real-life clinical setting as they under-represent population groups such as the elderly and those with co-morbidities, and exclude non-compliers, while critics of observational studies question conclusions drawn from studies that lack a control group and may be influenced by publicity and marketing.^28^

There are also conflicting findings on the question of whether the incidence of statin-associated myopathy varies with statin type and dose. A recent network meta-analysis of placebo-controlled trials and head-to-head trials comparing different statin types found small or non-significant differences in rates of adverse muscle events, and no clear dose relationship.^21^ In the large JUPITER trial of 20 mg rosuvastatin, there was no difference in the incidence of myopathy between those on the drug and those on placebo.^29^ In contrast, a survey of adverse events reported to the FDA found that the frequency of reports of muscle symptoms aligned with the relative potency of statin class, with the most frequent reports for rosuvastatin, intermediate risks for atorvastatin and simvastatin, and the lowest incidence of muscle symptoms for pravastatin and lovastatin.^30^

Several studies have implicated high-dose simvastatin (≥40 mg) in risk of myopathy: an incidence of approximately 0.4%-1% for definite myopathy and 0.1% for rhabdomyolysis has been reported for intensive therapy with 80 mg simvastatin.^1^^,^ 8 ^,^ 9 ^,^ 16 ^,^ 31 A further 1-2% of patients on high-dose simvastatin, and 0.2% on low-dose (20 mg) simvastatin, experienced moderate or incipient myopathy.^9^ In the large observational PRIMO study of high-dose statin therapy, almost 20% of patients on high-dose simvastatin reported adverse muscle symptoms, with low to intermediate incidence for atorvastatin and pravastatin, and the lowest risk for fluvastatin.^25^

The reasons for the discrepancies among studies on statin dose and type are not clear. The differing problems associated with clinical trials and observational studies have already been mentioned. Large prospective trials directly comparing statins at equipotent doses in well-defined patient populations, and with standardised definitions for myopathy, will probably be required to resolve the question.

A range of risk factors have been reported for statin-associated myopathy, including female gender (though not in all studies), low body mass index, older age, intense physical exercise, comorbidities such as hypothyroidism, and – most importantly – interactions with other drugs such as amiodarone and fibrates.^5^ In candidate gene studies, polymorphisms in a variety of genes affecting statin pharmacokinetics or pharmacodynamics have also been reported to influence statin-induced myopathy (reviewed in 32).

In 2008, a genome-wide association study (the SEARCH Study) found an association between myopathy on high-dose (80 mg) simvastatin and a polymorphism in the *SLCO1B1 *gene encoding organic anion transporting polypeptide 1 (OATP1B1), a membrane transporter that facilitates hepatic uptake of statins.^8^ The association was with a non-coding single nucleotide polymorphism (SNP) in *SLCO1B1 *that is in near-complete linkage disequilibrium with a functional SNP, rs4149056 (c.521T>C). At this locus, the wild-type T allele encodes valine while the minor C allele encodes alanine. The association between the rs4149056 (c.521T>C) C allele and simvastatin-induced myopathy has been independently replicated by subsequent studies.^33^^,^ 34 ^,^ 35 ^,^ 36 In addition, a genome-wide association study has shown an association between the C allele and rhabdomyolysis resulting from therapy with cerivastatin, which was withdrawn from the market in 2001 because of increased risk of this adverse reaction.^37^

The frequency of the C allele is less than 3% in some African populations, 10%-20% in European and Middle Eastern populations, 10%-15% in East Asian populations, 5%-10% in South/West Asian populations, and over 20% in some populations from South and Central America.^38^^,^ 39 The C allele occurs in the context of four different haplotypes: SLCO1B1*5 (rs4149056 alone), *15, *16 and *17.

The rs4149056 polymorphism has been shown to influence statin pharmacokinetics in all of these haplotypes: in studies on simvastatin, CC homozygotes (0%-6% of patients), with the lowest OATP1B1 activity, had higher plasma exposure to active simvastatin acid than TT homozygotes (55%-88% of patients); TC heterozygotes (11%-36% of patients) had intermediate exposure.^38^^,^ 40 It is thought that the higher plasma exposure to simvastatin acid in those with the C allele is responsible for myopathy, but the mechanism for the effect is not clear. Statins have been shown to cause muscle toxicity in vitro, with cerivastatin and simvastatin showing the strongest toxic effect, intermediate toxicity for fluvastatin and atorvastatin, and much lower toxicity for rosuvastatin and pravastatin.^41^

The effect of rs4149056 on statin pharmacokinetics also varies with statin type. Relative to the wild-type TT genotype, homozygosity for the C allele increases the observed plasma area under the curve by 221% for active simvastatin acid, 162%-191% for pitavastatin, 144% for atorvastatin, 57%-130% for pravastatin, 62%-117% for rosuvastatin and 19% for fluvastatin.^38^ Clear evidence for an effect of rs4149056 genotype on statin-associated myopathy has been reported so far only for high-dose (80 mg/day) simvastatin and cerivastatin, though there have been fewer studies on other statins. One study found an association between rs4149056 genotype and mild intolerance to atorvastatin in patients with familial hypercholesterolaemia^42^ but other studies have found no effect of this polymorphism for atorvastatin.^33^^,^ 34 ^,^ 36 ^,^ 43 Two studies, including the JUPITER trial, have found no effect of rs4149056 on myopathy in patients taking rosuvastatin.^29^^,^ 42

The SEARCH genome-wide association study found no evidence for significant associations between other *SLCO1B1 *SNPs and simvastatin-induced myopathy, but in haplotypes with rs4149056 there was an association of borderline significance (P=0.03) between the G allele of the functional variant rs2306283 (c.388A>G) and lower risk of myopathy.^8^ The effect of the rs2306283 polymorphism on the transport function of OATP1B1 is variable in different studies and may be substrate-specific.^44^ In some studies it has been found to be associated with slightly enhanced reduction in LDL-c in response to simvastatin.^8^^,^ 45 Donnelly et al. have reported that in diabetic patients, overall tolerance for simvastatin was affected by the combined genotype at rs4149056 and rs2306283.^35^ Puccetti et al. found no statistically significant effect of the rs2306283 G allele on rosuvastatin-induced myopathy in patients with familial hypercholesterolaemia^42^ and Santos et al. found no statistically significant effect on patients with familial hypercholesterolaemia who experienced myopathy with atorvastatin treatment.^43^ As evidence for an effect of rs2306283 on statin-induced myopathy is inconclusive at present, it will not be considered further in this paper.

The evidence for an association between the *SLCO1B1 *rs4149056 variant and simvastatin-induced myopathy has led to suggestions that *SLCO1B1 *genotyping might be used clinically (together with other risk factors) to guide choice and/or dose of statin therapy, with the aim of reducing risk of myopathy and improving adherence to therapy.^38^^,^ 46 Despite the observed effect on statin pharmacokinetics, evidence for an effect of *SLCO1B1 *polymorphisms on the efficacy of statin therapy is inconclusive.^47^^,^ 48 ^,^ 49 ^,^ 50 ^,^ 51 Results from the very large Heart Protection Study (18,000 participants on 40 mg simvastatin) suggested at most a small effect of *SLCO1B1 *genotype (approximately 1% per C allele) on LDL-c reduction and no significant effect on vascular outcomes.^8^^,^ 45

*SLCO1B1 *genotyping in the context of simvastatin therapy is already undertaken in some US institutions.^38^ For example at Duke University Center for Personalized Medicine, rs4149056 genotyping, together with guidance and advice to patients and providers, is offered for patients who have experienced simvastatin intolerance or are non-adherent to statin therapy. Alternative therapies are suggested for those with the *5 haplotype (rs4149056 alone), for example rosuvastatin if high potency is required, pravastatin if lower potency is acceptable, or fluvastatin if it is necessary to minimise interaction with other drugs.

Vanderbilt University Medical Center is piloting a pre-emptive pharmacogenomic strategy in 10,000 patients at high risk of coronary events.^52^ Patient genotypes for variants implicated in response to clopidogrel, warfarin and simvastatin are stored in their electronic medical record. The prescribing physician is alerted to this information at the point of prescribing and provided with a decision support algorithm to inform the choice of drug and/or dosing regimen.

## Test description

The rs4149056 variant can be tested on its own by a PCR-based SNP assay, or multiplexed on an array-based platform.^38^ There are commercial array platforms that include this variant, usually as one of a set of PharmADME pharmacogenetic variants, with genotyping by a proprietary assay.^38^ Genotyping services in CLIA-approved laboratories are available from several commercial suppliers and genotyping is also carried out by some clinical and academic institutions.^38^ **** *SLCO1B1 *SNP genotyping is included within some direct-to-consumer testing services.^53^

## Public health importance

Coronary heart disease is a major cause of morbidity and mortality in the industrialised world and, increasingly, in low- and middle-income countries. Introduced in the late 1980s, statins have become the most important pharmaceutical intervention for primary and secondary prevention of this condition, decreasing cardiovascular events by approximately 25%.^1^^,^ 3 ^,^ 4 In 2005-2008, 20% of adult Americans between the ages of 45 and 64, and 44% of those aged 65 and over (approximately 30 million in total) were taking statins.^54^ Data from the IMS Institute (www.imshealth.com) suggest that numbers of prescriptions have increased by 20% since 2007, so the current figure is likely to be about 36 million. Simvastatin is 2nd on the list of most commonly prescribed drugs in the US, with 96.6 million prescriptions for generic simvastatin written in 2011. In addition there were 40.8 million prescriptions for top-selling branded statin Lipitor® (atorvastatin) and approximately 20 million for Crestor® (rosuvastatin) (data from IMS Health; "The Use of Medicines in the United States: Review of 2011").

Although the frequency of statin-related myopathy is low, particularly for severe or life-threatening symptoms and at standard doses, the very high usage of statins means that the number of individuals affected by definite or incipient myopathy may be substantial: approximately 108,000 if an overall 0.3% risk is assumed for standard-dose therapy. However, accurate estimation is very difficult, for the reasons discussed above. Concerns about myopathy contribute to non-adherence to statin therapy,^55^ which is associated with higher all-cause and cardiovascular mortality.^56^ The frequency of non-adherence is greater at higher statin doses;^57^ in two studies of high-dose statin use, 9-10% of patients reported experiencing myalgia and, of those, 16-20% discontinued treatment, suggesting that 1.5-2% of patients on high-dose statin may discontinue therapy as a result of muscle symptoms,^25^^,^ 58 thereby increasing their risk of coronary events unless alternative lipid-lowering therapy is undertaken.

## Published recommendations and guidelines

- **FDA dosing recommendation**: In 2011, the FDA published a safety announcement recommending limiting the use of high dose (80 mg) simvastatin because of increased risk of muscle damage, which “*appears to be higher during the first year of treatment, is often the result of interactions with other drugs, and is frequently associated with a genetic predisposition towards simvastatin-related myopathy*” (http://www.fda.gov/Drugs/DrugSafety/ucm256581.htm). The announcement states that “*simvastatin 80 mg should be used only in patients who have been taking this dose for 12 months or more without evidence of muscle injury (myopathy). Simvastatin 80 mg should not be started in new patients, including patients already taking lower doses of the drug*.” The announcement also lists contraindications for simvastatin in combination with 11 other drugs, and dose limitations for simvastatin in combination with a further 6 drugs.
- **Final conclusions and Recommendations of the National Lipid Association Statin Safety Assessment Task Force **(2006)^11^ : These recommendations state that if muscle symptoms or elevated CK levels occur in a patient on statin therapy, other aetiologies should be ruled out before assuming a causal effect of the statin. A pre-treatment CK measurement may be considered in patients at high risk of myopathy. CK levels should be monitored during treatment in symptomatic patients in order to inform clinical decisions about continuing treatment. Treatment should be discontinued in patients who develop intolerable myopathy, regardless of CK levels; once symptoms have ceased, therapy may be continued with the same or a different statin at the same or a lower dose. If symptoms continue to recur, other lipid-lowering therapy should be considered. Patients who have tolerable muscle symptoms or who are asymptomatic with CK<10×ULN may continue therapy at the same or a reduced statin dose unless symptoms worsen. If a patient develops rhabdomyolysis (CK>10,000 IU/L or CK>10×ULN with elevated serum creatine or requiring intravenous hydration), statin therapy should be discontinued and the patient treated in hospital.
- **ACC/AHA/NHLBI Clinical Advisory on Use and Safety of Statins **(2002)^10^ : These guidelines recommend baseline CK measurement, with follow-up measurement if the patient reports muscle symptoms. If CK elevation is absent or moderate (3-10×ULN), advice should be given on avoiding over-strenuous activity, with weekly follow-up of symptoms and CK levels until symptoms resolve or worsen. Statin therapy should be discontinued if CK levels rise above 10×ULN; if symptoms resolve, therapy may be reinitiated at a lower dose if possible. Physicians should be aware of the risk factors for myopathy (particularly comedications) and take them into account in prescribing decisions.
- **The Clinical Pharmacogenomics Implementation Consortium Guideline for *SLCO1B1* and Simvastatin-Induced Myopathy **(2012)^38^ : The CPIC guideline is the only guideline specifically relating to *SLCO1B1 *variants. It recommends that prescribing physicians should be alerted to the FDA advice on avoiding high-dose simvastatin, and on contraindications for simvastatin prescription. If rs4149056 genotype is available and simvastatin is not contraindicated, the physician should consider adjusting simvastatin therapy according to the patient’s risk as indicated by their genotype: TT genotype (normal myopathy risk): Avoid 80 mg dose unless already tolerated 12 months. Prescribe desired starting dose and adjust doses based on disease-specific guidelines. TC genotype (intermediate myopathy risk): Avoid 80 mg dose. Consider a lower dose; if suboptimal efficacy, consider an alternative statin. CC genotype (high myopathy risk): Avoid 80 mg dose. Prescribe a lower dose or consider an alternative statin; consider routine CK surveillance. The guideline also includes a decision support algorithm.

## Evidence overview

**Analytic validity**

Pulley et al. have published evidence regarding the test performance of the Illumina VeraCode ADME microarray panel, which assays 184 pharmacogenetic variants in 34 genes and is genotyped by a specific version of Illumina’s GoldenGate® assay.^52^ In validation tests before the test was implemented clinically, the average concordance rate for 54 control cell lines with genotypes tested by “several other technologies” was 99.58%. Both a positive and a negative control are included in every 96 well plate. The performance of the assay for the *SLCO1B1 *locus is not stated specifically by Pulley et al, but it is not listed among loci displaying lower-than-average concordance. The manufacturer’s technical data sheet for the VeraCode ADME array assay claims 99.8% reproducibility between runs and 99.8% concordance with Sanger sequencing or whole-genome genotyping (http://www.illumina.com/documents/products/datasheets/datasheet_veracode_adme_core_panel.pdf).

For the Affymetrix DMET™ Plus array and proprietary DMET™ Plus genotyping assay, the manufacturer claims sample concordance to reference of ≥99.5%, reproducibility of ≥99.8% and sample pass rate of >95% (http://www.affymetrix.com/estore/browse/products.jsp?productId=131412#1_1).

Other published papers report a variety of assay methods for *SLCO1B1 *SNP analysis, including PCR-based TaqMan assays for single SNPs^35^ and re-sequencing of *SLCO1B1 *exons^8^^,^ 33 but provide little specific information related to analytic validity. Most studies report that the genotyped variants were in Hardy-Weinberg equilibrium.^8^^,^ 33 ^,^ 35 ^,^ 36 ^,^ 43 Santos et al carried out genotyping by PCR amplification followed by high-resolution melting analysis; results in some samples (number not specified) were verified by sequencing and 15% of samples were re-analysed, with 100% reproducibility.^43^ None of the case-control studies reported whether researchers carrying out genotyping were blind to case/control status.

**Clinical validity**

The strongest support for an association of statin-induced myopathy with the *SLCO1B1 *rs4149056 C allele comes from the SEARCH study of patients with prior myocardial infarction who were taking 80 mg simvastatin.^8^ In a genome-wide association study of 85 patients of European ancestry with “definite” or “incipient” myopathy and 90 matched controls, the C allele was associated with myopathy with an odds ratio of 4.5 per C allele (95% CI 2.6-7.7). The odds ratio was 16.9 (95% CI 4.7-61.1, P=2x10^-9^) for CC homozygotes relative to TT homozygotes. An even stronger association was found between the C allele and “definite” myopathy (OR for CC vs TT 27.2 [CI 6.8-109.2]). The cumulative risk of myopathy over five years of treatment with 80 mg simvastatin was estimated to be approximately 18% for CC homozygotes, 3% for TC heterozygotes and 0.6% for TT homozygotes, with 80% of the risk occurring during the first year of treatment, and 60% of myopathy cases on 80 mg simvastatin attributable to the rs4149056 variant. Importantly, the association between the C allele and myopathy was replicated in the large Heart Protection Study (HPS) of patients on 40 mg simvastatin, though with a smaller odds ratio of 2.6 per allele (95% CI 1.3-5.0, P=0.004).^8^

Significant associations between the C allele at rs4149056 and simvastatin-associated myopathy (variously defined) have also been reported in a retrospective case-control study of lipid clinic outpatients,^34^ a prospective clinical trial of patients with raised cholesterol,^33^ a retrospective case-control study of patients recruited through the UK Clinical Practice Research Datalink^36^ and (for CC homozygotes in a recessive model) in a retrospective cohort study of diabetics.^35^(Table 2). Overall, the studies provide support for an association between the C allele at rs4149056 and increased susceptibility to simvastatin-induced myopathy, with odds ratios between 2.1 and 4.7 per copy of the allele.

**Table 2. Studies on the association between the rs4149056 SNP and statin-induced myopathy:** 

Study	Statin type	Dose	Result
SEARCH Link 2008^8^	Simva.	80mg	OR 4.5 [CI 2.6-7.7] per C allele OR 16.9 [CI 4.7-61.1] for CC homozygotes, P=2x10^-9^ OR 4.7 [CI 3.5-6.4] P=3x10^-28^ if HPS controls used as control group
Heart Protection Study Link 2008^8^	Simva.	40mg	OR 2.6 [CI 1.3-5.0] per C allele, P=0.004
STRENGTH Voora 2009^33^	Atorva.	10mg 8 wks, 80mg 8 wks	OR 1.6 [CI 0.7-3.7]^a^
	Simva.	20mg 8 wks, 80mg 8 wks	OR 2.8 [CI 1.3-6.0]^a^
	Prava.	10mg 8 wks, 40mg 8 wks	OR 1.0 [CI 0.4-2.6]^a^
Brunham 2012^34^	Simva.	10-80mg (mean 30mg)	OR 3.2 [CI 0.83-11.96] P=0.042 for CC genotype OR 2.3 per C allele
		Atorva. 10-80mg (mean 30mg)	OR 1.06 [CI 0.22-4.80], P=0.48
Go-DARTS Donnelly 2011^35^	70% simva. 20% atorva. 5% fluva. 5% prava.	10-160mg	OR 2.05 [CI 1.02-4.09], P=0.043 for CC genotype in logistic regression for intolerance. Highest risk at ≥40mg simvastatin: OR 3.16 [CI 1.40-7.09] P=0.0055 for CC genotype in logistic regression for simvastatin
Puccetti 2010^42^	Atorva.	20-40mg	OR 2.7 [CI 1.3-4.9] P<0.001
	Rosuva.	20mg	OR 0.65 [CI 0.24-1.01] P=0.099
Santos 2012^43^	Atorva.	20-80mg	OR 2.24 [CI 0.47-10.72] P=0.31 for myalgia OR 1.51 [CI 0.57-3.96] P=0.41 for elevated CK
Marciante 2011^37^	Ceriva. (64% also on Gem-fibrozil). Controls on a range of statins		OR 1.89 [CI 1.40-2.56] P=3.62x10^-5^ per C allele OR 4.34 [CI 1.86-10.10] for CC genotype Stronger association for gemfibrozil users: OR 2.45 [CI 1.61-3.75] P=3.11x10^-5^ per C allele
JUPITER Danik 2013^29^	Rosuva.	20mg	HR 0.95 [CI 0.79-1.15] P=0.59 per C allele for myalgia HR 0.89 [CI 0.78-1.02] P=0.09 per C allele for muscle weakness,stiffness or pain
Carr 2013^36^	Cases: 78% simva. 14% atorva. 8% other statin Controls: 60% simva. 30% atorva. 10% other statin	Cases: mean dose 33.2mg Controls: mean dose 30.6mg	**All statins (n=448)** All myopathy OR 2.08 [CI 1.35-3.23] P=0.005 per C allele; OR 4.32 [CI 1.82-10.43] for CC vs TT Severe myopathy OR 4.47 [CI 1.84-10.84] P=0.0003 per C allele; OR 19.98 [CI 3.38-117.50] for CC vs TT **Simvastatin (n=281)** All myopathy OR 2.13 [CI 1.29-3.54]P=0.014 <40mg OR 1.03 [CI 0.45-2.36] P=0.997 ≥40mg OR 3.23 [CI 1.74-5.99] P=0.0002 Severe myopathy OR 4.97 [CI 2.16-11.43] P=0.0004 <40mg OR 1.84 [CI 0.34-9.86] P=0.778 ≥40mg OR 6.28 [CI 2.38-16.60] P=0.0004 **Atorvastatin (n=121)** All myopathy OR 1.91 [CI 0.56-6.54] P=0.613

^a^Taken from discussion of the STRENGTH study data by Wilke et al.^38^

Table 2 also summarises results from recent studies on the effects of rs4149056 genotype on myopathy with other statins (see Appendix for details of study type, population and outcome measures for these studies).^29^^,^ 33 ^,^ 34 ^,^ 35 ^,^ 36 ^,^ 37 ^,^ 42 ^,^ 43 A meta-analysis of seven studies on myopathy with any statin gave an overall odds ratio of 2.18 [CI 1.39-3.43] per C allele.^36^ For studies reporting specifically on simvastatin, the combined odds ratio was 3.25 [CI 1.72-6.12]. By contrast, carriage of the C allele gave a combined odds ratio for myopathy with atorvastatin that was not significant: 1.54 [CI 0.80-2.97].

From calculated values presented for the SEARCH study for the cumulative risk of myopathy associated with taking 80 mg simvastatin daily over 5 years (Figure 3 in reference 8, and using their estimate of 0.15 for the population prevalence of the C allele in the SEARCH population), the calculated sensitivity, specificity, positive and negative predictive values (PPV and NPV) for a test based on rs4149056 genotype for myopathy risk over 5 years of 80 mg simvastatin treatment are:

For ≥1 C allele: Sensitivity 70.4%, Specificity 73.7%, PPV 4.1%, NPV 99.4%.

For the CC genotype: Sensitivity 25.1%, Specificity 98.3%, PPV 18.6%, NPV 98.8%

Despite the high attributable risk for the *SLCO1B1 *rs4149056 polymorphism reported in the SEARCH study, other genetic polymorphisms are likely to contribute to risk of statin-associated myopathy, including rare variants not detected by genome-wide association studies.^32^^,^ 59 ^,^ 60 Overall response will be determined by a combination of multiple genetic and non-genetic factors, including gender, age, racial/ethnic background, BMI, co-morbidities and other medications. Recently a risk prediction algorithm has been developed for statin-induced myopathy (defined as a diagnosis of myopathy or rhabdomyolysis or CK≥4×ULN) in a UK population.^61^ The Qstatin risk score for myopathy (www.qintervention.org), which has been independently verified, though so far only in another UK population,^62^ has been found to have an Area Under the Receiver Operating Characteristic Curve (AUROC) of 73.9%-76.1% in women and 71.7%-72.7% in men for predicting 5-year risk of statin-induced myopathy; the percentage of explained variation (*R^2 ^*statistic) is 42% in women and 38% in men. In calibration analysis, the model was found to be reasonably well calibrated, but to over-estimate myopathy risk slightly in older age groups. In general, tests with an AUROC of less than 75% are not considered clinically useful,^63^ so the Qstatin score is at the borderline by this criterion. If addition of *SLCO1B1 *genotype could be shown to increase the AUROC of Qstatin significantly (at least for simvastatin), this would be an important contribution towards demonstrating good clinical validity for a combined test.

## Clinical utility

Direct demonstration of clinical utility would require evidence that using *SLCO1B1 *genotype to inform statin therapy (either before therapy is initiated or to guide management of those who have already experienced statin intolerance) results in net clinical benefit to patients: lower rates of myopathy together with adequate efficacy and acceptability of alternative treatment(s).

Although genotype-directed prescribing advice for simvastatin is currently offered at two institutions,^38^^,^ 52 there do not appear to be any published studies to date that compare clinical outcomes in patients whose statin prescription was directed or not directed by *SLCO1B1 *genotype. Now that the FDA advises avoiding 80 mg simvastatin in all patients except those already tolerant for at least 12 months, it may be difficult to demonstrate clinical utility for a test-and-treat prescribing strategy at lower simvastatin doses.

Some evidence exists that is relevant to clinical utility. Firstly, there are effective LDL-c-lowering alternatives to treatment with high-dose simvastatin, the statin therapy most clearly associated with risk of myopathy in carriers of the *SLCO1B1 *C allele. For example, fluvastatin, pravastatin and lovastatin may all have adequate efficacy if low potency is acceptable (reviewed in 5 ^,^ 6). Atorvastatin and rosuvastatin may be effective in high-risk patients requiring greater LDL-c-lowering potency.^5^^,^ 29 However, myopathy has also been observed with these statins in some studies.^23^^,^ 25 ^,^ 30 ^,^ 42 ^,^ 64 As both atorvastatin and rosuvastatin have long half-lives, non-daily dosing may reduce adverse effects while still achieving adequate reduction of LDL-c.^65^ Overall, approximately 20%-40% of patients with statin-induced myopathy are able to tolerate an alternative statin.^5^^,^ 26 As an alternative to statin switching, a lower dose of simvastatin may be combined with another lipid-lowering drug such as ezetimibe or colesevalam, or reinitiation of therapy on a low statin dose after symptoms have eased may be followed by gradual up-titration of the dose.^6^

In view of the clinical benefits that have been demonstrated for intensive statin therapy,^1^^,^ 9 direct evidence of good clinical outcomes in patients switched to or initiated on less intensive therapy as a result of *SLCO1B1 *genotyping would be desirable. Given the modest positive predictive value of the CC genotype but the much higher negative predictive value, the test might have greater clinical utility for selecting those (ie TT homozygotes) who are more likely to be able to tolerate higher-dose simvastatin, especially if they are at high risk of major coronary events (and assuming that higher doses do not cause other adverse effects).^9^ For CC homozygotes, who have an increased risk of myopathy, a statin therapy that has slightly lower efficacy may nevertheless result in net clinical benefit if it lowers the risk of non-adherence as well as the risk of rare, but serious, rhabdomyolysis. Evidence is sparse on this issue but some studies have found a higher rate of non-adherence in carriers of the *SLCO1B1 *C allele.^33^^,^ 35 It has been suggested that knowledge of the *SLCO1B1 *genotype may be particularly useful to inform prescribing and on-therapy safety monitoring if statins are to be used in conjunction with other drugs, and during the first year of therapy when the absolute risk of myopathy is highest.^8^

Westbrook et al. have pointed out the potential consequences, for both patients and clinicians, of incidental findings resulting from testing with pharmacogenetic SNP arrays.^66^ The rs4149056 polymorphism in *SLCO1B1 *has been found to be associated with uptake or clearance of other drugs including irinotecan and lopinavir, and there are reports that other *SLCO1B1 *variants are associated with risk of gallstone disease, hypertension and coronary artery disease.^38^ However, reported correlations of *SLCO1B1 *genotype with clinical phenotypes other than response to statins have yet to be independently replicated.^66^ Thus at present it appears that potentially harmful or confusing incidental findings related to *SLCO1B1 *genotyping are likely to be few.

The designers of the PREDICT project at Vanderbilt University Medical Center (VUMC) have undertaken pilot work to assess patients’ understanding and acceptance of use of pharmacogenomic tests in prescribing.^52^ 84% considered such tests acceptable though only 20% regarded them as “routine”. Most patients thought verbal consent to testing was sufficient, rather than formal written consent. The consensus with regard to incidental findings was that notification should be a matter of patient choice.

**Costs and cost-effectiveness**

No specific information was found relating to health-economic evaluation of *SLCO1B1 *genotyping in the context of statin prescription. Statin switching has cost implications if the switch is from generic simvastatin to a more expensive branded alternative such as Crestor® (rosuvastatin). However the overall cost implications of switching are likely to be small in comparison to other (largely nonclinical) factors affecting statin prescription.^67^ Better-targeted prescribing could lead to less drug wastage as well as decreased costs for medical consultations, tests and morbidity associated with statin-induced myopathy, but such effects have yet to be documented and quantified.

In a general discussion, the VUMC PREDICT group have set out the financial, organisational and professional commitment required to establish and run their pharmacogenomic prescription advice framework linked to patients’ electronic medical records.^52^ They estimate a total cost of $5 million over the first two years. They comment that they are seeking to establish payer reimbursement for genetic testing where a requirement for testing can be justified by FDA labelling advice. If pharmacogenetic testing with an array platform has already been established at an institution, there is essentially no marginal testing cost incurred by beginning to make use of additional variants within the same array, but each additional variant may still incur some costs in terms of education and support for prescribing physicians (and possibly also for patients), quality control and audit.

## Gaps in current evidence

The most important current gap in the evidence base is the lack of direct evidence on clinical outcomes resulting from prescribing decisions guided by *SLCO1B1 *rs4149056 genotyping and the lack, to date, of studies investigating the clinical validity of combining *SLCO1B1 *genotype with other risk factors for myopathy such as those included in the Qstatin risk score. The possibly protective effect of the rs2306283 (c.388A>G) G allele on statin-induced myopathy may merit further investigation, particularly in haplotypes where it occurs with the rs4149056 C allele (*15, *16 and *17).

Clarification is also needed on the relationship between risk of statin-induced myopathy and statin type and dose, and on whether the association between the rs4149056 polymorphism and risk of myopathy is specific for high-dose simvastatin, as current evidence suggests.

A further important deficiency is the lack of studies on population groups with ancestries other than white European. Allele frequencies at rs4149056 are known to vary in different populations but the effect of this variation on clinical outcome is not yet clear.^38^ The FDA recommends a lower maximum dose of rosuvastatin in patients of East Asian ancestry because of evidence of increased plasma exposure and greater lipid-lowering efficacy at lower doses in this population, but this effect does not appear to be attributable to *SLCO1B1.* 68 In the Qstatin risk score, racial/ethnic background is an important contributor to risk of statin-associated myopathy: risk is significantly higher in Caribbean and Black African groups (hazard ratios approximately 4 for women and 7 for men compared to white Europeans).^61^ However, this population has one of the lowest frequencies for the rs4149056 C allele,^38^ suggesting that the relative contribution of the rs4149056 variant to risk of statin-induced myopathy may vary in different racial groups.

## Appendix

**Characteristics of studies on effects of SLCO1B1 rs4149056 polymorphism on statin-induced myopathy:** 

Study	Type	Population	Outcome measure
SEARCH Link 2008^8^	Retrospective case-control	Patients and controls from SEARCH population with prior myocardial infarction (total populn 5761). European ancestry 85 cases, 90 controls 5-8y follow-up. C allele freq. in whole SEARCH populn 15%	"Definite" myopathy: muscle symptoms and CK>10×ULN "Incipient" myopathy: CK>3×ULN and >5×baseline plus AAT>1.7×baseline, irrespective of muscle symptoms
Heart Protection Study Link 2008^8^	Patients and controls with pre-existing occlusive vascular disease or diabetes	24 cases (21 genotyped), 16,643 controls. 5y follow-up	
STRENGTH Voora 2009^33^	Prospective, randomised open-label study	452 outpatients with hypercholesterlaemia. 16wk follow-up. 85% Caucasian, 5% African-American, 9% other race C allele freq 28%	Composite adverse event of CK>3×ULN or myalgia or discontinuation for any side-effect
Brunham 2012^34^	Retrospective case-control	Lipid clinic patients (patients and controls), Dutch ancestry. Simvastatin: 12 cases; 39 matched controls Atorvastatin: 10 cases; 39 controls	"Severe" myopathy defined as CK>10×ULN
Go-DARTS Donnelly 2011^35^	Retrospective cohort study	4196 Type 2 diabetics (1275 tolerant, 816 intolerant, 2050 unclassifiable). UK (Scotland) populn C allele freq 16.2%	Mild intolerance defined as composite of CK 1-3×ULN or abnormal AAT (if no abnormality at baseline) *and* "relevant prescribing change"
Puccetti 2010^42^	Observational case-control	Patients and controls with familial hypercholesterolaemia. European ancestry. 76 cases (46 atorva., 30 rosuva.); number of controls not stated	Mild intolerance defined as myalgia/muscle cramps, or CK>3×ULN
Santos 2012^43^	Retrospective case-control	Patients and controls with familial hypercholesterolaemia. Brazillian: 87% Causasian, 10% mulatto, 3% African. At least 12 months follow-up. 14 cases, 129 controls	Mild intolerance defined as myalgia/muscle cramps irrespective of CK values, or CK>3×ULN regardless of symptoms
Marciante 2011^37^	Retrospective case-control	Patients with severe rhabdomyolysis, pooled statin-using controls (matched by sex) from Heart and Vascular Health Study and Cardiovascular Health Study. 185 cases, 732 controls	Severe rhabdomyolysis
JUPITER Danik 2013^29^	Case-control study within randomised placebo-controlled trial	Participants with no prior cardiovascular disease or diabetes, with LDL-c<130mg/dL and C reactive protein≥2mg/L. European ancestry. 4404 on statin; 4378 on placebo	"Clinical myalgia" or a broader definition of muscle weakness, stiffness or pain
Carr 2013^36^	Retrospective case-control	Patients recruited via UK Clinical Practice Research Datalink. 77 cases, 372 controls	Myopathy: CK>4×ULN Severe myopathy: CK>10×ULN, and/or rhabdomylysis
